# Evaluation of the effects of photobiomodulation on orthodontic movement of molar verticalization with mini-implant: A randomized double-blind protocol study

**DOI:** 10.1097/MD.0000000000019430

**Published:** 2020-03-27

**Authors:** Felipe Murakami-Malaquias-Silva, Ellen Perim Rosa, Paulo André Almeida, Tânia Oppido Schalch, Carlos Alberto Tenis, Renata Matalon Negreiros, Ricardo Fidos Horliana, Aguinaldo Silva Garcez, Marcella Ueda R. Fernandes, Andre Tortamano, Lara Jansiski Motta, Sandra Kalil Bussadori, Anna Carolina Ratto Tempestini Horliana

**Affiliations:** a Postgraduate Program in Biophotonics Applied to Health Sciences, Universidade Nove de Julho, UNINOVE; b Academic specialization student in Temporomandibular Disorder and Orofacial pain, Universidade Nove de Julho, UNINOVE; c São Leopoldo Mandic, School of Dentistry, Campinas; d Coordinator of Graduation course; e Department of Orthodontics, School of Dentistry, University of São Paulo, São Paulo, Brazil.

**Keywords:** dental implants, low-level light therapy, orthodontics interceptive, tooth movement techniques

## Abstract

**Introduction:**

Loss of a dental element can generate several repercussions in the stomatognathic system. According to the latest survey by the Ministry of Health, in 2010, Brazilian adults had, on average, 7 missing teeth. This loss may lead to movement of the adjacent teeth and the antagonist, which would make prosthetic rehabilitation harder to do. Anchoring systems, such as mini-implants, have been increasingly used as a treatment option because they act with heavy but controlled forces and without side effects. Recent studies have shown that photobiomodulation (PBM) can accelerate orthodontic movement in molar intrusion. The objective of this study will be to evaluate the effect of PBM on the acceleration of the orthodontic movement of molar verticalization and its effect on pain and inflammation of the periodontal tissues.

**Patient concerns:**

the concerns assessments will be done over the study using anamnesis interviews and specific questionnaire.

**Diagnosis:**

verticalization will be evaluated by clinical and radiographic analysis.

**Interventions:**

Thirty four healthy patients aged 30 to 60 years, who need to recover the prosthetic space for oral rehabilitation after loss of the posterior inferior dental elements and inclination of the adjacent element, will be randomly divided into 2 groups: G1 (control group) - verticalization by mini-implant + PBM simulation (placebo); G2 (experimental group) - verticalization by mini-implant + PBM. The movements will occur with the aid of mini-implants and elastomeric chains ligatures. The PBM will occur with diode laser application, 808 nm, 100 mW, receiving 1J per point, 10 seconds, 10 points (5 per buccal and 5 per lingual) and radiant exposure of 25 J/cm^2^. The orthodontic forces of verticalization (corresponding to any exchange of elastomeric ligation) will be applied every 30 days and the PBM will be applied immediately, 3 and 7 days of each month, for a period of 3 months. The crevicular gingival fluid (CGF) will be collected on the 1st, 3rd, and 7th days after the first activation, and then on the 3rd day of the following 2 months.

**Outcomes:**

Interleukins IL1β, IL-6, IL-8, IL-10, and TNF-α will be analyzed by ELISA. Panoramic radiography will be performed at baseline and 90 afterwards to ascertain the amount (in degrees) of verticalization. To evaluate the pain, the Visual Analog Scale (VAS) will be used in all the consultations, and to evaluate the quality of life, the Oral Health Impact Profile (OHIP-14) questionnaire will be applied. Analgesics will be given and the quantity of drugs will be counted. If the data are normal, they will be submitted to Student *t* test. The data will be presented as means ± SD and the value of p will be defined as <0.05.Discussion: This protocol will determine the effectiveness of photobiomoduation regarding the orthodontic movement of molar verticalization.

**Ethics and dissemination:**

This protocol received approval from the Human Research Ethics Committee of *Universidade Nove de Julho* (certificate number: 3 533 219). The data will be published in a peer-reviewed periodical.

## Abstract 2

Supplemental Digital Content is available in the text

## 1 Introduction

In clinical practice, we often come across mesially inclined molars, whether due to an early loss of deciduous or permanent molars (from caries, periodontal disease, trauma), ectopic eruption, and second premolar anodontics, which results in an adjacent teeth impaction (second and third molars). In such cases it is common to find infraosseous defects in the mesial of the inclined molar and a reduction of the interradicular space in the distal region.^[1]^

One of the solutions for the prosthetic rehabilitation of this space would be the verticalization of these molars with opening or closing of the space or extraction in more severe cases, such as very severe angles or mobility of the element.^[1]^

However, this isolated vertical movement is very difficult to perform, especially with the most common clinical routine techniques, such as the use of springs and cantilevers,^[1]^ and is usually accompanied by an extrusion, which often produces premature contacts, and bite opening, interfering with the entire occlusion. A mechanical control with well-defined theoretical bases, with minimal undesirable effects, would be ideal.^[1]^

Verticalization of molars with skeletal anchorage consists of the dislocation movement of these teeth distally, without reactions in the most anterior segments of the arch.^[2]^ It is an interesting device because it is transitory, with skeletal anchorage (which does not allow the movement of the reaction unit) and fast results, with controlled forces.^[3]^ According to Roberts et al 1990^[4]^ and 1996,^[5]^ the skeletal anchorage movement rate of the second and third molars is approximately 0.5 mm / month, which corresponds to the linear rate of osteoclastic resorption.^[6]^

For the molar vertical movement, the mini-implants should preferably be installed distally to the disinclination direction, close to the occlusal plane, thus reducing the intrusive vector in the molar mesial and, consequently, its inclination.^[3]^ For lower molars, mini-implants should be placed in the retromandibular region.^[3]^ By placing a fixation attached to the molar, where possible (occlusal, distal, or mesial face), orthodontic activation can be performed through closed springs, tie wires or elastic bands.^[3]^

Nowadays, with the ease of access to orthodontic treatment, and with the new therapeutic possibilities, there has been an increase in adult demand for faster, more effective treatments with as few side effects as possible. These same adults are the ones who by 2010 had a toothless rate of about 7.4 teeth,^[7]^ requiring not only isolated orthodontic movement, but complete oral rehabilitation.

As an option, several studies have reported different methods for accelerating this orthodontic movement, which can be divided into 3 categories: biological, through local, or systemic drug administration (local injections of prostaglandins or administration of muscle relaxants); Mechanical or physical stimulus (vibratory forces, photobiomodulation, and laser); and surgical access to facilitate movement (corticotomies).^[8,9,10]^

The administration of systemic substances has been thought of in animal studies,^[11]^ as prostaglandin E2 and calcium gluconate, but human studies are still scarce and little explored, requiring further studies to assess their cost-effectiveness if they really affect dentoalveolar tissues and your safety. Local injections of drugs, vibration, or electrical stimulation, besides being able to cause pain and discomfort, would still require specific equipment and materials, just for this use, that is not so common in daily clinical practice. Added to this is the fact that the surgical treatment is an invasive procedure, with difficult acceptance by the patient.^[9]^

However, some care must still be taken. With excessive forces, there is a physiological decrease in the amount of tooth movement due to a decrease in vascularization in the compression areas, and an increase in bone density. In these cases, angiogenic stimulating effects of photobiomodualtion (PBM) may be beneficial for these patients.^[12,13,14]^

In this scenario, the use of PBM using laser or LED^[15]^ irradiation has been widespread, with good receptivity on the part of the patients, being easy to apply, painless, with minimal adverse effects and applied to different areas of dentistry.^[16–19]^

PBM has important analgesic, anti-inflammatory, biostimulatory, and tissue repair effects,^[20,21]^ that is, accelerating the processes of cell multiplication and regeneration, provided that the laser is used within the proper parameters.^[22]^ In pain, PBM acts on the production of betaendofirna (the body's natural pain reduction mediator),^[23]^ and inhibits the production of aracdonic acid^[24]^ (which, by generating metabolites such as prostaglandins and interleukins which bind to nociceptors, cause pain) and the number of nociceptive transmitters responsible for conducting nerve impulses.^[25]^

The modulatory action on tissues is the result of the ability of PBM to accelerate metabolic changes, increasing bone resorption and neoformation, essential for orthodontic movement to occur.^[26]^

In the inflammatory process generated by tooth movement, various cytokines, chemokines, and growth factors can be detected in crevicular gingival fluid (CGF).^[27]^ During orthodontic tooth movement, an increase in the expression of inflammatory mediators is expected,^[28,29]^ such as interleukins (IL). They are released by various cell types residing in the periodontal ligament, such as fibroblasts and osteoclasts, and these cytokines are responsible for bone remodeling and modulation of the inflammatory response. The most studied are IL-1β, IL-6, IL-8, IL-10, and TNF-α.^[30,31,32]^ Interleukin IL-1β is important because it stimulates osteoclastic activity and recruits leukocytes and other cellular mediators that contribute to the process of bone remodeling. Giannopoulos et al^.^^[33]^ and Yao et al^[34]^ observed an increase in IL-1β levels in relation to tooth movement. In turn, IL-6 is responsible for regulating local inflammatory immune responses and stimulating osteoclast formation and bone resorption activity.^[35]^ The presence of IL-8 stimulates osteoclast differentiation in orthodontically moved tooth compression zones.^[36]^ While TNF-α is considered a pro-inflammatory cytokine, and its increase stimulates osteoclastic activity to the detriment of an inhibition of osteoblast activity and apoptosis of osteocytes.^[37]^ Finally, IL-10 is considered an anti-inflammatory cytokine, and its presence indicates a late phase of inflammation. When found at elevated levels, there is a decrease in IL-1β, IL-6 and TNF-α release and osteoclastogenic activity.^[38]^ In order to evaluate cell dynamics, CGF sampling is a noninvasive and non-destructive method of choice.^[39]^

Given the importance of seeking balanced occlusion, it is necessary to correct the position of vertical molars in adult patients. Currently, mini-implants have enabled the correction of a dental element alone. Therefore it is very indicated in adults, however it is still necessary to accelerate the treatment time and modulate the inflammation bringing a less painful postoperative period with lower consumption of analgesics. In this sense, photobiomodulation has been used with good results in small orthodontic movements of adult patients such as molar intrusion. There was less pain and less production of inflammatory cytokines, indicating that PBM modulates the inflammatory response and accelerates movement. Although molar verticalization is a common movement among adult patients, so far no clinical studies have evaluated pain inflammation, movement rate, and quality of life of patients. Therefore, the present study aimed to evaluate the effects of PBM on orthodontic movement of vertical molars by evaluating the expression of proinflammatory cytokines, pain reduction, movement speed, and patients quality of life.

## 2 Methods

This is a prospective, single-center, randomized, controlled, double-blind, 90-day follow-up trial that meets the criteria for a SPIRIT Statement clinical trial design. This protocol received approval from the Human Research Ethics Committee of *Universidade Nove de Julho* (certificate number: 3 533 219). Any complications or alterations will be reported and clarified to REC and reported in the publications. After verbal and written explanation of the study (FMMS), patients who agree to participate in the study will sign the Informed Consent Form (ICF). If patients want to receive the research data, they can put the e-mail in the informed consent form and the full article can be checked as soon as it is published. The study will be conducted in accordance with the Helsinki Declaration (revised Fortaleza, 2013). The Project was registered at www.clinicaltrial.gov with NCT 04037709. The patients will come from Dra. Fernanda C. Dias's office and staff, located at Avenida Mariana Ubaldina do Espirito Santo, 761 – Bom Clima, Guarulhos/SP, Brazil. There is no conflict of interest with regard to the clinics where the research will be conducted and with respect to any product used at work and with respect to any author involved in the study. Project receive grant from Brazilian National Coordination for the Improvement of Higher Education Personnel CAPES #690822 (CAPES Portuguese: Coordenação de Aperfeiçoamento de Pessoal de Nível Superior (CAPES).

The sample will consist of adult patients, who live in the city of São Paulo, Brazil, and who require lower molar verticalization for prosthetic rehabilitation. The description will be detailed in the item “Orthodontic verticalization mechanic and placement of the mini-implant”. The experimental design will consist of 2 groups (will be described in detail in the item “Experimental Design”). Samples will be collected for evaluation of inflammatory cytokines of crevicular gingival fluid. The intensity of pain will also be evaluated through the visual analog scale, the amount of medication taken and the vertical velocity of both groups. An OHIP-14 questionnaire will also be applied to measure oral health-related quality of life (Fig. 1  flowchart). Data will be published and there will be no restriction on including data for publication. All data will be available for consultation, and all patients will have access to their records at any time.

**Figure 1:**
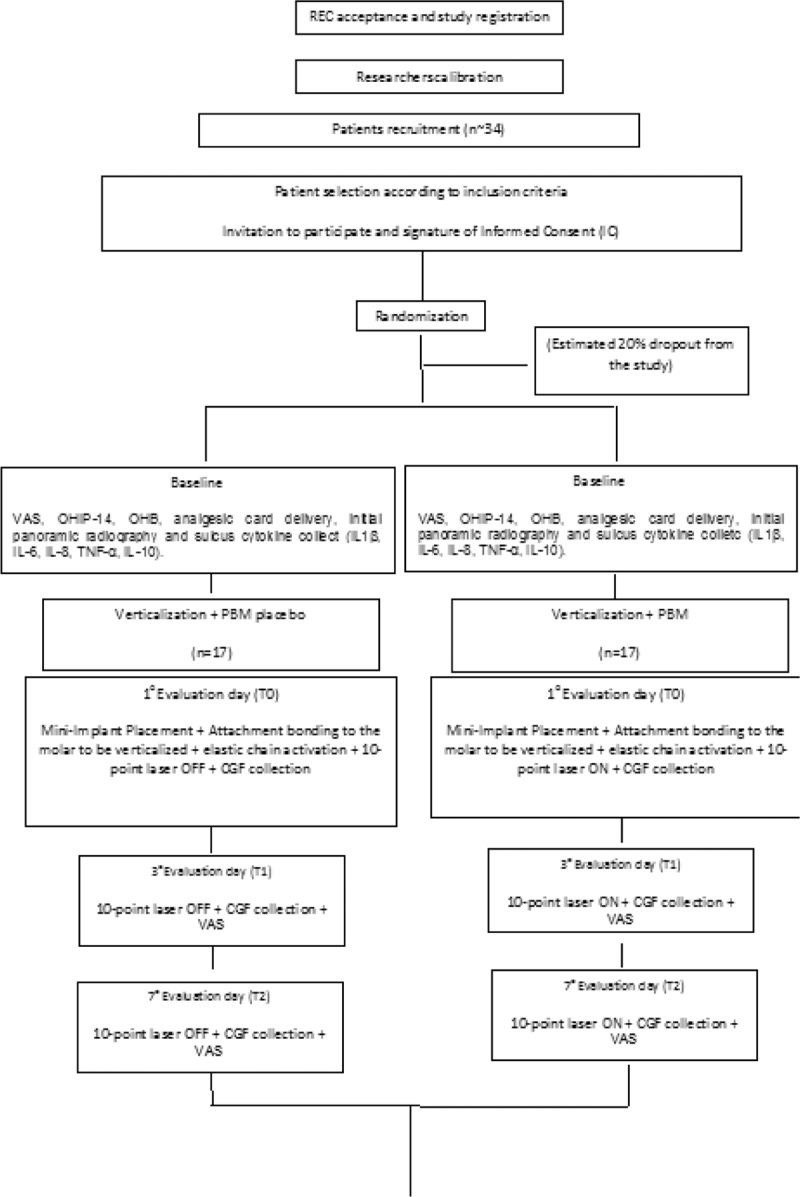
Flowchart.

**Figure 1 (Continued):**
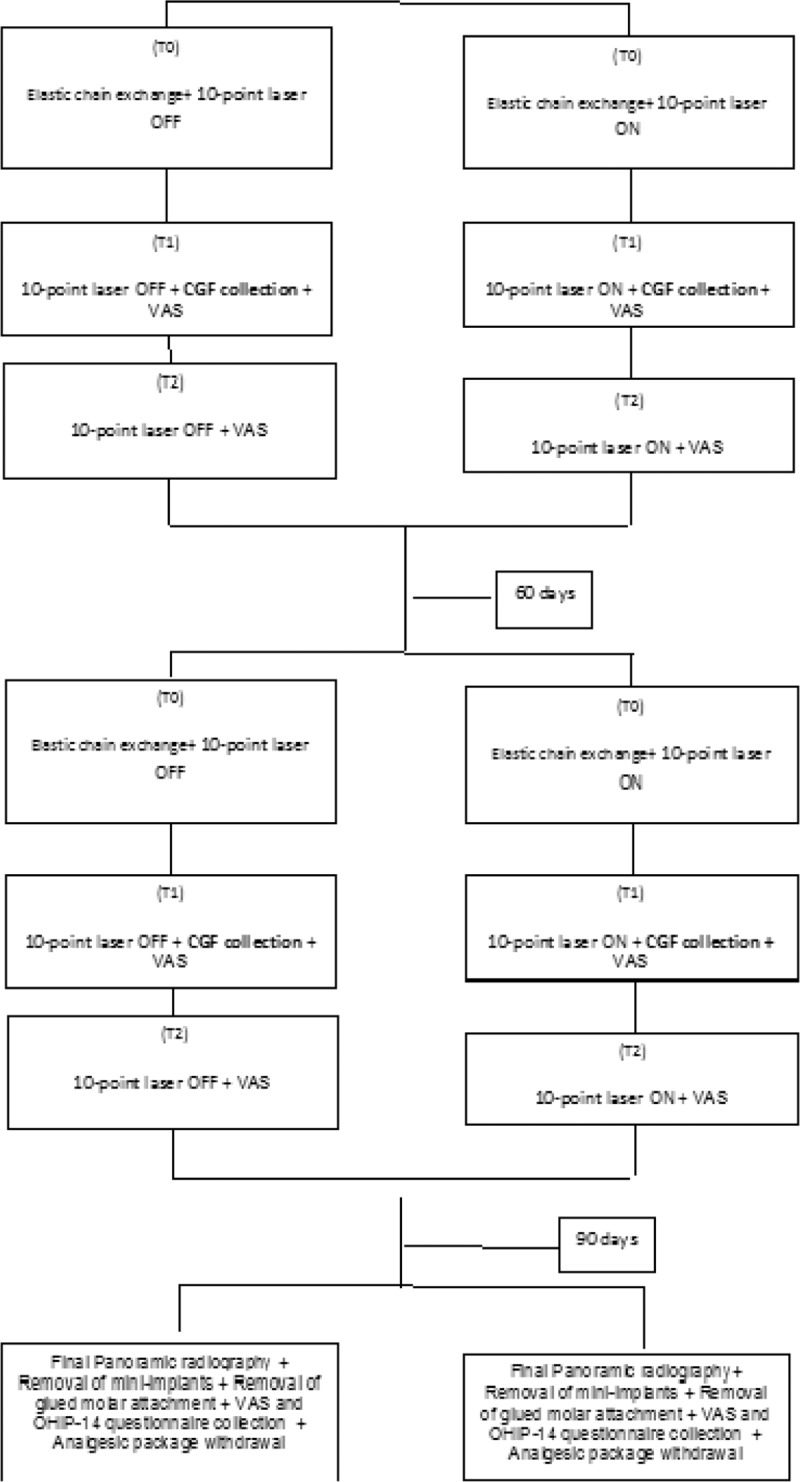
Flowchart.

### 2.1 Sample size calculation

To achieve an effect size = 0.40 between 2 groups (Orthodontic verticalization mechanic and Orthodontic verticalization mechanic + PBM), to increase the molar verticalization velocity assuming the type 1 error of 0.2^[40]^ and 95% confidence level, the total sample size will be 34 patients, divided into 2 groups. The sample calculation was performed in Excel, based on the calculation formula described by Kadam and Bhalerao. Considering a 20% drop-out, 3 extra patients will be inserted into each group if there is dropout.^[41]^

### 2.2 Calibration of researchers

For calibration, an examiner, trained in dentistry and specializing in orthodontics, will evaluate 5 panoramic radiographies of inclined molars, which will not be part of the study. Two radiographic evaluations will be made (10 days apart) to check for intra-examiner agreement. The intraclass correlation coefficient (ICC) will be calculated to assess intra-examiner agreement ≥ 0.75 with respect to the measurement of lower molar inclination using pre-established radiographic measurements (described in item “Movement rate”). The evaluation will be performed with millimeter ruler + protractor and this researcher will not be involved in the treatment of patients. The same examiner will be trained to collect crevicular gingival fluid (CGF) for cytokine analysis and data collection for OHIP-14 to maximize reproducibility of evaluations.

### 2.3 Description of the sample

The sample will consist of patients aged 30 to 60 years, living in the city of São Paulo, Brazil, considered healthy after anamnesis and clinical evaluation, without periodontal disease, under orthodontic treatment or not, and needing recovery of the prosthetic space for oral rehabilitation, after lower molars loss and consequent inclination of adjacent elements. The procedures will not entail charges to patients, only to travel to the place of research (which, if necessary, may be supported by the researcher)

### 2.4 Inclusion/exclusion criteria

Patients who are latex allergic, pregnant or breastfeeding, smokers, diabetics, patients undergoing head and neck radiotherapy, coagulation disorders requiring antibiotic prophylaxis for placement of mini-implants, with absolute indication for use of local anesthetics with vasoconstrictors, with decompensate systemic disease, with systemic or local infection (periodontitis or periodontal abscess), who have used anti-inflammatory drugs in the last 3 months before orthodontic treatment, will be excluded from the search.

Healthy patients (ASA I - negative medical history), systolic blood pressure less than 140 mm Hg and diastolic blood pressure less than 90 mm Hg, heart rate with 70 ± 20 beats/minute, requiring oral rehabilitation after loss of some posterior lower dental element (1st molar), with favorable periodontal condition to the installation of mini-implants and who agree and sign the informed consent, will be included.

Patients who may have any complications during the research period, such as: allergic reactions to any of the materials used, allergic reaction to paracetamol, have taken any other drug than the one available, etc. Or those who are in any way unable to attend any of the appointments on the scheduled dates (in all, will be 10 dates, within 3 months), change cities, or simply no longer wish to participate in the survey, will be automatically excluded, without any compromise or damage to it (with removal of the installed apparatus). They will still be advised not to go to other professionals to intervene or continue treatment. Patients will be assisted by researchers for all research problems.

Any questions during the research period should be informed to the researcher so that the researcher can take the appropriate measures (the latter should provide some form of personal contact).

### 2.5 Randomization

To randomly assign participants to the experimental groups, a 40-number draw (34 patients + 20% of drop out, if need be) will be conducted using the Microsoft Excel 2017 version program. The distribution of the groups will be identical (1: 1) for both groups. Distribution will be performed in a blocked manner (5 groups of 8 patients). Opaque envelopes will be identified with sequential numbers (1 to 40) and inside there will be information from the corresponding experimental group according to the order obtained in the draw. The envelopes will be sealed and remain sealed in numerical order until the time of mini-implant placement and laser application. The draw and preparation of the envelopes will be performed by a person not involved in the study. Immediately before the laser application, the treating researcher will open 1 envelope (without changing the number sequence) and perform the indicated procedure. Three additional patients will be included in each group due to the drop out predicted in every clinical study (considering a 20% drop out). The author of the research will be responsible for the storage (digital platforms) and tabulation of information, being the statistician responsible for analyzing the data obtained at the end of the research. These same envelopes will contain the patient records (with anamnesis sheet, ICF and radiographs) and will be kept on file.

### 2.6 Study blinding

Only 1 external research collaborator, with dental training, who will be responsible for the treatments (which will open the envelopes of randomization), will know which photobiomodulation treatment will be assigned to each patient. The identification of each group will be revealed by this collaborator to all involved only after statistical analysis of the data. Therefore, the researcher responsible for data collection and the statistician will be blinded to the treatments assigned to the groups. The patient will also be blind to the type of treatment performed, since the mini-implant placement treatment will be identical in both groups and the laser treatment for photobiomodulation will be simulated in the control group.

### 2.7 Pretreatment evaluations

The patients will sign the Informed Consent Form Supplemental Digital Content (ANNEX 1) and the anamnesis Supplemental Digital Content (ANNEX 2) and the OHIP-14 questionnaire Supplemental Digital Content (ANNEX 3) will be applied. A form will be given to the patient to note the time and amount of medication taken. These data will be collected by 1 trained researcher (according to item.2.2 ’Researcher calibration’). Crevicular gingival fluid (CGF) will also be collected by this same professional. And also, the assessment of the impact of oral health on quality of life (OHIP-14). The visual analog scale will be applied immediately before the start of the procedure and will be asked about the amount of painkillers in the last week prior to the surgical procedure. Then the treatment will be performed as randomized by the researcher with dental training, specialist in orthodontics, responsible for the treatment.

### 2.8 Anamnesis

Anamnesis will be performed in both groups. In addition to questions related to the patient's general health, demographic data (age, gender, marital status, occupation, educational level, living conditions, salary), medical history data (major complaint, current disease status, medical history, dental history, medications).

### 2.9 Oral hygiene guidance (OHB)

All participants will receive oral hygiene guidance (OHB), Bass brushing technique, toothbrush specifications (soft bristles and small head), and dental floss (traditional Colgate)

### 2.10 Experimental design

Immediately before photobiomodulation (PBM) the responsible researcher will remove and open 1 envelope (without changing the numerical sequence of the other envelopes) and perform the indicated procedure. Thus, the 34 patients will be allocated to the experimental and control groups (considering a 20% drop out) as follows:

G1- Control group (n = 17) - Molar verticalization + laser simulation (placebo) - the treatment of molar verticalization adjacent to tooth loss will be performed with the use of mini-implants for skeletal anchorage (described in detail in the item “2.11 - Orthodontic verticalization mechanic and placement of the mini-implant”), followed by simulation of laser use, since it will be off, being applied at the same points and in the same period of the group that will be irradiated. To mimic the laser action the noise of the BIP will be recorded. Laser mimicking will occur immediately, 3 and 7 days after placement of the mini-implant, and the same sequence will be applied 30 and 60 days after force application (elastomeric ligature replacement), 5 points for buccal and 5 points for lingual. The analgesic pack will be delivered to the patient who will be advised to use only in case of pain. Visual analog scale (VAS), initial molar inclination (panoramic radiography), number of analgesics in the last 10 days, quality of life questionnaire (OHIP-14) and CGF collection will be assessed at baseline. Pain and use of rescue medication will be evaluated in all sessions. CGF collection will always be performed on the second day of laser application in the 3 months of research (totaling 3 collections, outside the baseline) and the questionnaire (OHIP) after 90 days. Another panoramic radiograph will be solicited before the removal of mini-implant (after 90 days).

G2- Experimental group (n = 17) Molar verticalization + PBM (n = 17 + 3) - the treatment of molar verticalization adjacent to tooth loss will be performed with the use of mini-implants for skeletal anchorage (described in detail in the item “2.11 - Orthodontic verticalization mechanic and placement of the mini-implant”), followed by the use of laser. Immediately 3 and 7 days after surgery, the same sequence was applied 30 and 60 days after force application (elastomeric ligature change), 5 points for buccal and 5 points for lingual, following the long axis of the tooth. The analgesic pack will be delivered to the patient who will be advised to use only in case of pain. Visual analog scale, initial molar inclination (panoramic radiography), number of analgesics in the last 10 days, quality of life questionnaire (OHIP-14) and CGF collection will be assessed at baseline. Pain and the use of rescue medication will be evaluated in all sessions. CGF collection will always be performed on the second day of laser application in the 3 months of research (totaling 3 collections, outside the baseline) and the questionnaire OHIP-14 after 90 days. Another panoramic radiograph will be requested before the removal of mini-implant (after 90 days). (Fig. 1 )

### 2.11 Orthodontic verticalization mechanic and placement of the mini-implant

At the first consultation, all patients will be clinically and radiographically evaluated, and a treatment plan will be drawn up. Given the need for molar verticalization, an orthodontic mini-implant (Morelli, Sorocaba, SP, Brazil) will be installed in the retromandibular region (Araújo, 2006). First there will be a local asepsis with 2% chlorhexidine solution (2% aqueous chlorhexidine - Rioquímica) and external asepsis with 0.2% chlorhexidine (0.2% aqueous chlorhexidine - Rioquímica). The site will be anesthetized with a carpule (quinelato), short gingival needle (Unoject - Nova DFL) and the anesthetic of choice will be Mepivacaine + 2% Epinephrine 1 ml (Mepiadre 2% 1: 100000 - Nova DFL). The anesthetic technique used will be the infiltrative (essentially local) at 3 points of the retromolar region (central, buccal and lingual). Once this is done, the region will be pre-drilled by about 4 mm with a spearhead coupled to a manual key (Morelli ). In order to have proper anchorage and primary stability, the dimensions of the mini-implant will be chosen based on panoramic radiography and the amount of cortical bone present in the region.^[42]^ Once the mini-implant has been selected (Morelli, Sorocaba, SP, Brazil), it will be locked into position using a kit-specific digital key (Morelli, Sorocaba, SP, Brazil). Being threaded clockwise until the intramucosal mini-implant is at the gingival level. A simple surgery, where there is no osseointegration of the mini-implant, being inserted only in the cortical bone, being therefore easy to remove, and can be activated (force application) immediately. After installation, a metallic orthodontic lingual button (Morelli, Sorocaba, SP, Brazil) will be glued to the molar mesial face to be verticalized. As the molar is verticalized, this lingual button may be retracted more distally so that the elastic always exerts the proper force (this will be evaluated at each patient return by the calibrated operator). For its installation, molar prophylaxis will be performed with a Robinson brush (Robinson straight CA - Microdont brush) and prophylactic paste (Herjos - Coltene), followed by water irrigation and drying. Once this is done, the dental surface will be conditioned with 37% phosphoric acid (Condac - FGM) for 45 seconds, followed by water irrigation for 15 seconds and subtle air drying from the triple syringe. Next, we have the application of single bond adhesive system (Adper Single Bond 2 - 3M Adhesive) and polymerization (Poly Wireless Curing - Kavo) for 5 seconds. The lingual button will be glued to the inclined molar mesial with orthodontic adhesive (Orthocem - FGM), and a small portion of it will be placed on the lingual button grid and, once it is in position, it will be light cured for 45 seconds. A gray chain elastomeric ligature (Morelli, Sorocaba, SP, Brazil) will attach the head of the mini-implant to the lingual button glued to the molar mesial. A light force of 150 g will be imposed (measured with a tensiometer - Morelli, Sorocaba, SP, Brazil)^[43,44]^ in all cases, regardless of tooth position. This chain elastic will be changed every 30 days for a period of 3 months. After 90 days of the experiment time, the mini-implants will be removed. This procedure involves local anesthesia with Mepivacaine + 2% Epinephrine 1 ml (Mepiadre 2% 1: 100000 - Nova DFL) and the mini-implant will be removed only with the digital key being turned counterclockwise. Since there is no osseointegration, its removal is simple and quick, requiring no incision or suture. Painkillers will be available if there is pain. Removal of the lingual button will be performed using bracket pliers (346R-Zatty Straight Bracket Orthodontic Pliers) and resin removal with high-speed diamond drill bits (FG – Fava flame diamond). After the trial period, for those patients who are interested will be offered the possibility to complete (if this 3 months of research has not been achieved) the full verticalization of this molar (ie the mini-implant and lingual button would not be removed after 90 days and changes of the elastic bandage would occur until the case was finalized), so that they could receive future oral rehabilitation (making it clear to the patient that they would no longer be part of the research, and that any charge resulting from this choice would not fit to the researcher).

### 2.12 Methodology for PBM application

**Figure 2:**
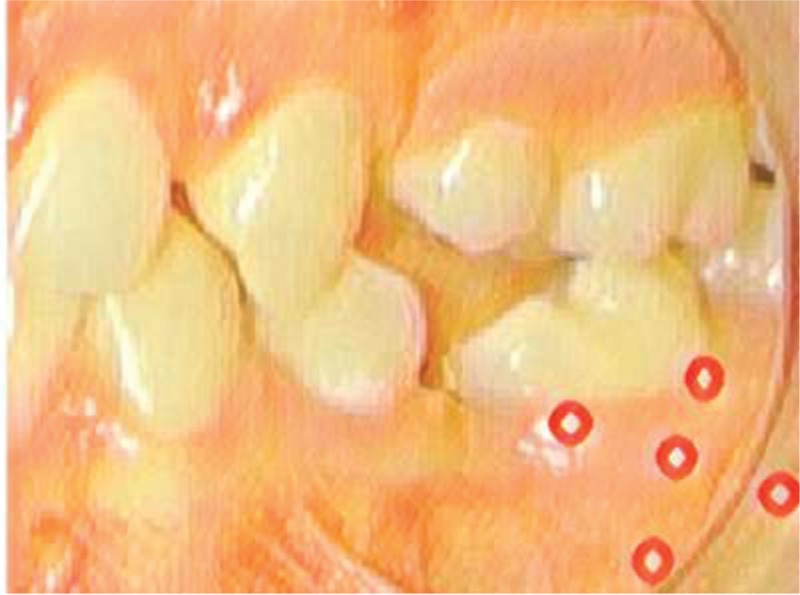
Laser application on buccal surface of the inclined molar.

Patients form Group 2 will receive laser treatment (photobiomodulation) in order to modulate orthodontic movement and act on inflammation and pain. The procedures will be performed immediately after the application of forces (placement of elastic bandages) on the tooth, as described:

- The irradiations will be performed with a diode laser emitting infrared wavelength (Therapy XT - ANVISA RDC Standard 185/2001 - DMC, São Paulo, SP, Brazil). The power output of this equipment is 100 mW and the wavelength used will be 808 nm (±10 nm). The optical fiber diameter of the device is 600 μm, therefore a spot (area) of 0.002826 cm^2^. The energy delivered per point will be 1J. This will require a irradiation of 10 seconds per point. As 10 points will be irradiated, the total application time will be 100 seconds and the total energy delivered will be 10J. The energy density will be 25 J/cm^2^ and the power density will be 35.38 W/cm^2^ (Table 1).^[40]^
- The laser tip will be positioned perpendicular to the edge in direct contact with the vertical tooth mucosa, 10 seconds per site: 5 points per buccal and 5 points per lingual: 2 points, mesial and distal, in what would be considered the cervical third of the root; a central point in the middle third, and 2 points, mesial and distal, which would be equivalent to the apical third of the root (Fig. 2).
- During laser application both, patient and operator, will wear security goggles adequate to laser wavelength.
- The laser will be applied immediately after orthodontic force application (T0), 3 (T1) and 7 (T2) days later. After 30 days, the elastomeric ligature will be changed (therefore, renewal of the vertical force) and this same protocol will be applied (at times T0, T1 and T2). This same elastic bandage change will also occur 60 days after the mini-implant installation, and again the laser application protocol will be followed. Finally, 90 days after baseline, the mini-implant will be removed, as will the lingual button of the molar mesial, but without further laser application.

**Table 1:**
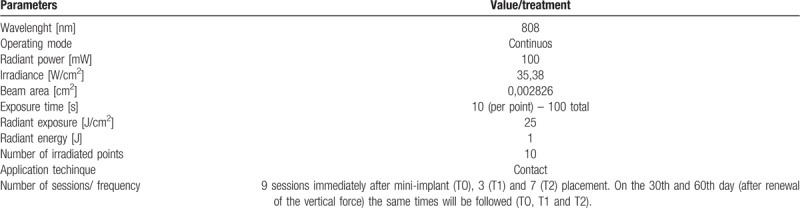
Photobiomodulation parameters.

Patients in Group 1 (Control) will receive the photobiomodulation placebo, that is, the same treatment performed in Group 2 to mimic the laser application, but with the laser off at the time of application. Group 2 procedures will be performed immediately after the application of forces (placement of elastic bandages) on the tooth, as described:

- Simulations will be performed with the same laser (Therapy XT - ANVISA RDC Standard 185/2001 - DMC, São Paulo, SP, Brazil). This will require 10 seconds of application simulation per point. As 10 points will be simulated, it will take 100 seconds for this simulation.
- The laser beep will be recorded to simulate its operation. Thus the patient will be blind to this procedure. The only person who will know the identity of the groups will be the person applying the laser. The rest of the researchers will not know the interventions made in the groups.
- The laser head will be positioned perpendicular to the lip in direct contact with the upright tooth mucosa. 5 points will be irradiated by buccal and 5 points by lingual, following the long axis of the tooth that will undergo the movement, being: 2 points in the cervical third, 2 points in the apical third, and 1 in the middle third (Fig. 2).
- During laser application both, patient and operator, will wear goggles.
- The laser will be applied immediately (T0) after the mini-implant and lingual button placement procedure, joined by the elastomeric chain ligation, 3 (T1) and 7 (T2) days later. After 30 days, the elastomeric ligature will be changed (therefore, renewal of the vertical force) and this same protocol will be applied (at times T0, T1 and T2). This same elastic bandage change will also occur 60 days after the mini-implant installation, and again the laser application protocol will be followed. Finally, 90 days after baseline, the mini-implant will be removed, as will the lingual button of the molar mesial, but without further laser application.

### 2.13 Study variables

The primary variable of the study will be

- Radiographic measurements to assess movement rate through tooth angulation vs time at baseline and the final of the treatment.

The secondary variables of the study will be:

- Oral health-related quality of life (HRQoL) will be assessed using the OHIP-14 baseline questionnaire applied at baseline and 90 days after treatment.
- Assess pain during orthodontic verticalization movement using the Visual Analog Scale (VAS) at baseline 30, 60, and 90 days after mini-implant placement
- Assess the amount of painkillers ingested in the period to verify that this therapy is effective in reducing pain during baseline orthodontic movement, 3 and 7 days, 30, 60, and 90 days after mini-implant placement.
- evaluation of IL-6, IL-1β, IL-8, TNF-α and IL-10 cytokines by baseline (T0), 3 (T1) and 7 days (T2), and then only in T1 (on 30 and 60 days).

*Movement rate* - With the aid of a ruler (Morelli, Sorocaba, SP, Brazil) and a protractor (transparent Waleu 10290001), an initial panoramic radiography will be compared to a final panoramic radiography (on day 90th) to ascertain the amount (in degrees) of verticalization. By draw a mandibular line (where 1 end would be the lower posterior part of the right goniaco angle region and the other its corresponding left side) and the long axis of the tooth,^[45]^ forming an angle (the larger, the larger the inclination of the tooth). These measurements will be recorded in degrees in each patient's chart for further analysis.

*Pain during movement* - Will be assessed by applying the visual analog scale (VAS) with a 100 mm line, with both ends closed. One end has the indication “0” and the other “100” which means “no pain” and “unbearable pain” respectively. Marking instructions will always be given to the patient by the same operator. Each patient will be instructed to mark with a vertical stroke the point that best corresponds to the pain intensity at the moment of evaluation.^[46]^

*Rescue medication* - Another parameter analyzed will be the amount of analgesics ingested, as proposed by Bauer 2013.^[47]^ At the beginning of the research, a paracetamol (drug with purely analgesic effect^[48]^) card will be delivered to each patient. It should be stored until the end and its use will be released only in case of pain. At the end of the experiment, the number of pills will be evaluated as another parameter to measure pain. This data will be collected by an auxiliary.

*Analysis of oral health-related quality of life (Oral health impact profile questionnaire - Ohip-14)* - This questionnaire is a simplified form of the original OHIP-49 questionnaire. Ohip-14 will be used to assess the impact of oral health on the quality of life of research participants.^[49]^ Ohip-14 is used to measure perceived needs. It measures the impact of oral changes on oral health related quality of life. The participant will answer 14 questions by giving their answers the values 0 (never), 1 (almost never), 2 (sometimes), 3 (most of the time) and 4 (always).

It can be analyzed from 2 perspectives:

- Additive method - Points are summed (0–56), with 56 being the biggest impact on quality of life.
- Domain Assessment - OHIP-14 has a total of 7 domains, which can also be scored from 0–4, with 4 being the biggest impact. To arrive at the result, the 2 questions of each domain are summed and divided by 2. Domain 1 refers to functional limitation, domain 2 refers to physical pain, domain 3 refers to physical limitation, domain 4 refers to psychological discomfort, domain 5 refers to psychological disability, domain 6 refers to social discomfort domain 7 refers to general disability (Table 2).

**Table 2-:**
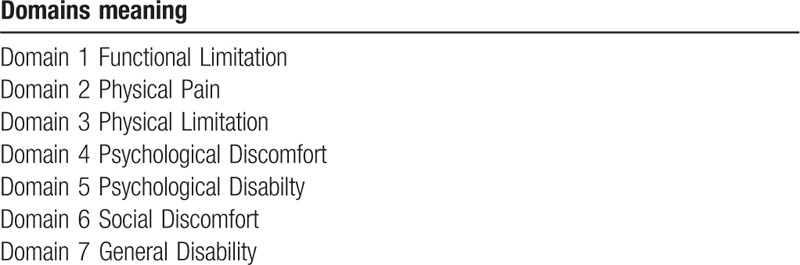
OHIP-14 main domain.

In this paper we will evaluate Ohip-14 by the additive method and domain evaluation. In the additive method, when the value is greater than 1, the negative impact on the individual's life can be considered. Ohip-14 will be evaluated at baseline and after 3 months for all individuals by an auxiliary.

*Collection and analysis of cytokine profile of crevicular gingival fluid* - Firstly, the biofilm present will be cured in the supra-gingival portion to avoid sample contamination. The site will be insulated with cotton rollers. A sterilized paper cone (#25 – Allprime cellpack, Brazil) will be inserted into the biological space of the vertical molar at 2 points: in the center of the buccal and lingual teeth until resistance is felt. The cone will be in position for 30 seconds. If there is contamination with blood should use new cone after 90 seconds. 10 cones should be collected, 1 for each cytokine, at the 2 analysis points. The cones will be placed in 1 sterile 1.5 ml micro centrifuge tube (Eppendorf, Sigma, CA, USA) and stored at −80° C.^[39]^ We will make another spare tube with 5 more cones in the same way as described. During collection will be stored on ice inside a properly identified Styrofoam. The collected samples will be stored at −80°C until further analysis and will be collected by an auxiliary.

*Determination of crevicular gingival fluid levels of the inflammatory markers* - TNF-α, IL1-β, IL-6, IL-8, and IL-10 will be performed by ELISA using commercial kits (Peprotech Inc., Rocky Hill, NJ, USA), according to manufacturer's instructions. This collection will be performed in the same periods of PBM application: before the placement of the mini-implant, immediately (T0), 3 (T1) and 7 (T2) days after, and at T1 of the 30th and 60th day.

### 2.14 Statistical analysis

Statistical analysis will be performed using only data obtained from patients who complete the study. The Kolmogorov-Smirnov test will be applied prior to data analysis to assess the normality of each parameter. Bidirectional analysis of variance (ANOVA) followed by Bonferroni test will be used to compare all measurements. All tests will be performed using Graphpad Prism 5 (GraphPad Software, San Diego, USA). The significance level will be set at p < 0.05.

## 3 Discussion

The study was characterized as a randomized, double-blind, placebo-controlled trial to minimize measurement bias (also referred to as information, observation or measurement), which is the result of systematic differences in the way data about the event of interest is obtained from the various groups under study.^[50]^

Randomization should be performed in prospective studies comparing the effect of an intervention (prophylactic or therapeutic) with their respective controls. The researcher distributes the intervention at random through randomization; Thus, the experimental and control groups are formed by a random decision process.^[50]^

The term double-blind refers to those trials in which neither the patient nor those responsible for their care and evaluation know what treatment he is receiving. Blinding will be performed by randomization during delivery of envelopes, and the group to which the patient belongs is confidentially informed on the brown envelope. The main reason for introducing a placebo-controlled group is to make patients’ attitudes as similar as possible to those of the treated group.^[50]^

For our study, the calculated sample was based on the “amount of motion x time” variable, following the Kadam, 2010,^[41]^ sample size calculation formula: N = 2 (ZA - Z1-β) 2σ2 / Δ2 reaching a “n” of 34 patients (17 patients in each group), with 20% dropout, totaling 3 more individuals in each group. Research participant dropout is unlikely during treatment because it has the mini-implant installed. Therefore, we limited our study to a 90-day follow up.

Our approach was based on a problem that can be pointed as one of the most recurrent conditions in the population, which is the loss of the permanent lower first molar.^[51,52]^ Of various causes, this situation can result in mesial migration of the second molars,^[53,54]^ affecting even the anterior teeth, with opening of diastemas and midline deviations.^[53,55]^ In such cases it is still common to find infraosseous defects in the mesial of the inclined molar, accompanied by a gingival recession and a reduction of the interradicular space in the distal region.^[1]^

One solution to this problem would be the disinclination of this molar, a movement known as verticalization. This movement returns the parallelism to the teeth that may later receive a fixed or removable denture.^[56]^ Thus, vertical bone defects are eliminated or reduced without the need for surgical intervention, enabling adequate edentulous space to be obtained for prosthetic rehabilitation and improved crown-root ratio in periodontally compromised molars.^[56]^

For this verticalization there are several techniques described in the literature, such as removable appliances, fixed appliances associated with continuous arcs, mechanical segmented with cantilever springs.^[1,57,58]^ Basically, tooth movement mechanics can be performed through 2 types of system: 1 statically determined and 1 statically undetermined.^[59]^ In the first 1, an action arm (cantilever) is supported at 1 end of the fixed appliance, and the other is free, allowing better control of forces and predictability of side effects. In the second system, both ends of the wire are supported on the fixed apparatus, which generates unpredictability both in the direction and magnitude of the force.^[60]^

It is clear to say that for all these types of movement there is a need for the installation of fixed apparatus for force control. Being by itself a long treatment, we can still face deformations of the metal arches, observed mainly by inadequate forces on them during feeding, leading to increased treatment time, deleterious effects on dental elements located at the extremities of the prosthetic space and lack of control in orthodontic movement.^[61]^ Anchorage control has been the biggest challenge in orthodontics.^[61]^

In order to circumvent these adverse effects, Kamomi, 1997^[62]^ and Costa et al, 1998^[63]^ proposed the use of mini-implants as orthodontic skeletal anchorage. As advantages, they are small enough to be placed in any area of the alveolar bone, have rapid healing and can be easily removed after use.^[64]^ We also have that orthodontic forces can be applied immediately after implantation,^[64]^ resisting movement when subjected to the load of this orthodontic force.^[65]^ It is an interesting alternative because it does not require patient cooperation,^[66,67,68,69]^ being useful in reducing treatment time, not requiring the installation of a fixed apparatus, minimizing or eliminating any kind of unwanted effect on natural teeth that would previously receive these forces during standard orthodontic treatment.^[61]^

However, some care must still be taken. Working with excessive forces, there is a physiological decrease in the amount of tooth movement due to a decrease in vascularization in the compression areas, and an increase in bone density,^[12,13,70]^ besides being able to generate displacement of the mini-implant,^[3]^ its stability must be conferred to each consultation and the forces applied in a controlled manner (based on the use of tensiometer). Stability may also be compromised due to peri-implant tissue inflammation, generated by poor hygiene,^[71]^ which would warrant thorough oral hygiene guidance.

In the search for a safer and more controlled action of this movement, Photobiomodulation (PBM) also appears as an adjuvant method in the treatment. Pain (observed during tooth movement) may act decreasing prostaglandin^[72]^ and inhibit aracdonic acid production.^[24]^ The modulatory action in tissues is the result of the ability of PBM to accelerate metabolic changes, angiogenesis and increase in the repair process directly linked to bone formation.^[72–74]^ Increased bone resorption and neoformation are essential for orthodontic movement to occur.^[26]^

When working with PBM, the wavelength commonly used and with positive results is around 780 nm^[74,75]^ and 808 nm,^[40]^ that is, in the infrared range, which is the longest. penetration into human tissues.^[24]^ Of the few studies that sought to approach PBM during orthodontic movement, 2 had promising results: Fernandes, 2019,^[40]^ who worked with intrusion movement and wavelength 808 nm. Fernandes 2019^[40]^ had the laser application occurring monthly, always after the application of the forces. This frequency in Fernandes, 2019,^[40]^ can be explained by the fact that the elastomeric chains undergo degradation and loss of almost 76% of the amount of force after 21 days.^[76]^ In addition, numerous studies have shown that the ideal strength range for orthodontic movement ranges from 100 to 450 g.^[61]^ Petrey, 2010,^[77]^ stated that in implants up to 6 mm, the point of failure starts at 461gr of force. To ensure greater stability of the mini-implant in posterior mandibular region, forces less than 2N (200grs) applied immediately and a diameter larger than 1.0 mm are indicated.^[78]^

To analyze the effectiveness of a new method, pain and cytokine analysis are important markers. As subjective as it may be, pain can be measured by counting the number of analgesics consumed during the research^[47]^ and by applying the Visual Analog Scale (VAS), being a way to better interpret and understand the patient's pain, facilitating care planning and decision making, making care more humane and responsive to patient needs.^[46]^

Cytokines, on the other hand, are important inflammatory markers, and as proposed by Kinney, 2014,^[39]^ can be obtained from the manipulated site through crevicular gingival fluid (CGF), which can be easily collected with the help of delicate stems and which carries biological molecular markers collected from the inflamed periodontium.^[39]^

Finally, to analyze the tooth movement rate, tracings made on an initial and final panoramic radiography (after 3 months) will be performed following the model proposed by Ursi, 1990,^[45]^ to analyze dental angulation. It draws a mandibular line (which does not change because it is based on the bony base rather than the occlusal plane) and the long axis of the tooth^[45]^ forming an angle (the larger, the larger the inclination of the tooth). This is the simplest, fastest, most affordable method with the least radiation exposure to the patient.^[45,79]^

## Author contributions

**Conceptualization:** Anna Carolina Ratto Tempestini Horliana, Felipe Murakami Malaquias da Silva, Sandra Kalil, Lara Motta

**Data curation:** Anna Carolina Ratto Tempestini Horliana, Felipe Murakami Malaquias da Silva, Tania Schalch

**Formal analysis:** Anna Carolina Ratto Tempestini Horliana, Felipe Murakami Malaquias da Silva, Renata Matalon Negreiros, Tania Schalch

**Investigation:** Anna Carolina Ratto Tempestini Horliana, Felipe Murakami Malaquias da Silva, Renata Matalon Negreiros, Carlos Tenis.

**Methodology:** Anna Carolina Ratto Tempestini Horliana, Felipe Murakami Malaquias da Silva, Andre Tortamano, Aguinaldo Garcez.

**Project administration:** Renata Matalon Negreiros, Anna Carolina Ratto Tempestini Horliana, Felipe Murakami Malaquias da Silva, Sandra Kalil.

**Resources:** Anna Carolina Ratto Tempestini Horliana, Felipe Murakami Malaquias da Silva, Ricardo Horliana.

**Supervision:** Renata Matalon Negreiros, Anna Carolina Ratto Tempestini Horliana, Aguinaldo Garcez, Andre Tortamano, Ricardo Horliana.

**Validation:** Anna Carolina Ratto Tempestini Horliana, Aguinaldo Garcez, Sandra Kalil, Lara Motta.

**Writing – original draft:** Anna Carolina Ratto Tempestini Horliana, Felipe Murakami Malaquias da Silva, Renata Matalon Negreiros

**Writing – review & editing:** Renata Matalon Negreiros, Anna Carolina Ratto Tempestini Horliana, Paulo Almeida, Ellen Perim Rosa do Nascimento, Tania Schalch

Anna Carolina Ratto Tempestini Horliana orcid: 0000-0003-3476-9064.

## Supplementary Material

Supplemental Digital Content

## Supplementary Material

Supplemental Digital Content

## Supplementary Material

Supplemental Digital Content
