# Supplementary material for: Evaluation of the effects of photobiomodulation on orthodontic movement of molar verticalization with mini-implant: A randomized double-blind protocol study
Source: Medicine (Baltimore). 2020 Mar 27;99(13):e19430. doi: 10.1097/MD.0000000000019430 (PMC7220149; doi:10.1097/MD.0000000000019430)
Supplement: Supplemental Digital Content [file medi-99-e19430-s001.docx]

**Annex 1: The Informed Consent Form**

ICF - Informed Consent Form for Participation in Clinical Research

Name:________________________________________________________

Address:__________________________________________________________________

Contact phone number: ______________________City:________________ CEP:______________

E-mail: ____________________________________________________________________

**1. Title of Experimental Research:**

Effects of laser on tooth declination (after loss of tooth in front of bottom teeth, molars) with the use of a mini-implant (“Evaluation of the effects of photobiomodulation on orthodontic movement of molras verticalization with mini-implants”).

**2. Aim:**

Evaluate how the laser acts during the dislocation movement of a tooth that has “laid down” towards the space left by a missing tooth.

**3. Justification:**

The loss of a tooth is common in the dental office. One of its consequences is that over time, the back tooth will begin to tilt (lie down) towards the empty space, preventing a new tooth from fitting into that space (either with the placement of an implant or a dental prosthesis). Therefore, this study will work with a method already used for the dislocation of these teeth and use the laser to evaluate if there is any improvement in this movement.

**4.**  **Experimental Phase Procedures:**

After evaluation and planning, selected participants will undergo a simple mini-implant surgery in the region behind the last tooth (about 40 minutes). These implants are easy to remove (temporary) and a small piece of metal will be glued to the inclined tooth. In the mini-implant and the small piece glued to the tooth, an elastic band will be placed that will make the force to dislocate the tooth that is lying down. Then, on the same day, a laser (light) will be applied to all participants' gums at 5 points on the cheek and 5 points on the inside facing the participant tongue (about 1 minute and 40 seconds per session). You should return after 3 days for a new laser application at the same points and at the same application time, and once again on the 7th day after the first application. Every 30 days, for a period of 3 months, the only procedure that will be performed will be to change the elastic for a new one, and 3 new laser applications (on the return day, 3 and 7 days after) will be done (totaling 10 visits to the office until the end of the research). 90 days after the mini-implant placement surgery, the mini-implant will be removed (about 20 minutes), as will the metal piece attached to the tooth that was tilted. All participants will be provided with a pain medication card (paracetamol®) released during the study period, as well as a questionnaire on quality of life and pain level. an initial (full-mouth) panoramic x-ray will be taken at the beginning of the survey, and a final x-ray will be taken on the day of mini-implant removal (90 days after its installation). And in laser applications, a little saliva near the gum will be collected with a paper straw (4 minutes total) for future analysis. At the end of the research all collected materials will be discarded.

**5**. **Discomfort or Expected Risks:**

Mini-implant placement surgery is relatively simple and will not be unlike any surgery routinely done in dental offices. You will be anesthetized locally (all patients who have any contraindications to anesthetic use will be excluded) and a pain medicine card will be made available to you for free. We will ask you to use the medicine only if there is pain, and do not take the medication unnecessarily. Rejection of the mini-implant material as well as the elastic material is quite rare, but if it does, you will be removed from the research without prejudice to their treatment. Laser use is painless, with no side effects and no contraindication. If you have questions or feel the need for a different medication you can contact us 24 hours on the phones placed below. A questionnaire will be applied to find out if there was an improvement in their quality of life after the treatments. You may feel embarrassed with some questions. We will ask about your pain (you will have to rate your pain from 0 to 10). We'll ask you about the amount of painkillers you took and if there was a need.

**6. Risk protective measures:**

Use of local anesthetics to avoid pain and availability of pain medication (analgesic - paracetamol®) if necessary. The questionnaire will be answered in a private room.

**7. Research Benefits:**

Tilting one tooth into the space left by the loss of another tooth is quite common, and prevents placement of a replacement tooth in place. Therefore, methods that improve, accelerate and make this movement more comfortable for the participant should be studied. Thus giving back quality of life, aesthetics and function. If you join the group with laser simulation, there will be no direct benefit to you from participating in this research, only for people who do this treatment in the future.

**8. Existing Alternative Methods:**

Currently, other alternatives to dislocate this tooth would be the use of springs or handles of various types and techniques to push this tooth, but all of these require the installation of fixed orthodontic braces to happen. The technique used in this work, no, is much less bothersome and practically painless.

**9. Withdrawal of Consent:**

All applicants have complete freedom to withdraw from the survey at any time without prejudice. Since the procedures performed so far can be undone without any commitment to the participant.

**10. Confidentiality guarantee**

All data collected will be stored in the records of each participant and can be checked at any time. All results and data derived from this research are for scientific purposes only.

**11. Forms of Compensation for Expenses arising from Participation in Research:**

All participants are free of charge.

**12. Research location:**

The research will be conducted in a private dental office, the Clinic of Dr. Fernanda Dias and Staff, located at Avenida Mariana Ubaldina do Espirito Santo, 761 - room 2 and 3, Bom Clima, Guarulhos, Sao Paulo, Brazil - CEP: 07197-000 . Phone: (11) 2443-2689

Responsible: Dra. Fernanda Caetano Dias

**13.** Research Ethics Committee (CEP) is an interdisciplinary and independent collegiate body, which should exist in institutions conducting research involving human subjects in Brazil, created to defend the interests of research participants in their integrity and dignity and to contribute to the development of research. Within the ethical standards (Standards and Regulatory Guidelines for Research on Human Beings - Res. CNS No. 466/12 and Res. CNS 510/2016). The Ethics Committee is responsible for the evaluation and monitoring of research protocols regarding ethical aspects.

Uninove Ethics Committee Address: Rua. Vergueiro nº 235/249 - 12th floor - Liberdade - São Paulo - SP ZIP code. 01504-001 Phone: 3385-9010 comitedeetica@uninove.br

Ethics Committee office hours: Monday to Friday - 11:30 am to 1:00 pm and 3:30 pm to 7:00 pm

**14.**  **Researchers (Advisor and Students)** **Full Name and Contact Phones:**

Prof. Dr. Anna Carolina Ratto Tempestini Horliana - (013) 98199-9848,

PhD student Felipe Murakami Malaquias da Silva - (011) 99647-8650.

**15**. Any complications that may arise during the research may be discussed by their own means.

Sao Paulo, from of .

**16**. **Post-Informed Consent:**

I, ________________________________________________, after reading and understanding this term of information and consent, understand that my participation is voluntary, and that I can leave the study at any time, without prejudice. I confirm that I have received a copy of this consent form, and authorize the research work and the dissemination of data obtained only in this study in the scientific community.

_______________________________

Participant Signature

(All sheets must be initialed by the research participant)

**17**. I, ________________________________________ (Researcher responsible for this research), certify that:

a) Considering that research ethics implies respect for human dignity and the protection due to participants in scientific research involving human beings;

b) This study has scientific merit and the team of professionals properly cited in this term is trained, qualified and competent to perform the procedures described in this term;

                                          ____________________________________

(Felipe Murakami Malaquias da Silva)

Responsible Researcher's Signature
