# Supplementary material for: Evaluation of the effects of photobiomodulation on orthodontic movement of molar verticalization with mini-implant: A randomized double-blind protocol study
Source: Medicine (Baltimore). 2020 Mar 27;99(13):e19430. doi: 10.1097/MD.0000000000019430 (PMC7220149; doi:10.1097/MD.0000000000019430)
Supplement: Supplemental Digital Content [file medi-99-e19430-s002.docx]

**Annex 2: Anamnesis form**

Anamnesis Form

| Date: ____/_____/_____ _ Patient Id: |
| --- |
| Researcher: |

Name:_______________________________________________ Id:_____________________

Date of Birth: ___/___/_____ Native Nationality: _________ occupation: _______________

Ethnicity: ( ) Leuco. ( ) Melano. ( ) Others: _______ Genre ( ) M ( ) F

Estado Civil: Married ( ) Single ( ) Others: __________

Education Level: Fundamental ( ) High School ( ) College ( ) Complete ( ) Incomplete ( )

Life Conditions: Own House ( ) Rented ( ) Others: __________ rooms number ( )

Private car ( ) Public transportation ( )

Address: ___________________________________________________________________

District: ____________________________________ City: ___________________

Phone number: _________________________________________

Medical history:

( ) **Hypertension** ( ) Kidney Problems ( ) Rheumatic Fever

( ) Hipotension ( ) Gastritis ( ) Endocarditis Inf.

( ) **Cardio Problems** ( ) Gastric Ulcer ( ) Hemophilias

( ) **Cardio surgery**  ( ) Hepatitis __ ( ) Haemorrhage

( ) AVC (Cerebral Vascular Accident) ( ) Tuberculosis ( ) Transplants

( ) **Diabetes** ( ) Sífilis ( ) Convulsion

( ) thyroid Problems ( ) HIV positive ( ) epilepsy

( ) Psychiatrists Treatment ( ) Childhood illnesses: ______________________

Have or had any unreported health problems? __________________________________________________________________________________________________________________________________________________________

Are you currently undergoing medical treatment? No ( ) Yes ( ) which one?______________

Do you use any medicines for continuous use? ____________________________________

Do you have any allergies? No ( ) Yes ( ) Which? _______________________________

Are you or have you been in medical / dental treatment in the last 3 months? Yes ( )No ( )

Pregnant or lactating? No ( ) Yes ( )

Have you used anti-inflammatory drugs in the last 3 months? No ( ) Yes ( ) which?__________________________________

Dental Issues Main complaint: _____________________________________________

Dental History: ____________________________________________________ ______________________________________________________________________

How long was your last visit to the dentist? __________________________

Have you ever had dental anesthesia? No ( ) Yes ( ) Did you have any reaction? __________________________

General Condition: PA _____________Heart Rate ________ Weight height _______

Harmful Habits / Habits

How many times a day do you brush your teeth? __________ Do you floss? Yes ( ) No ( )

If so, how often? Always ( ) Occasionally ( ) Almost Never

Ethylism? Yes ( ) Quantity / day ___________ No ( ) How long have you stopped? _________

Smoking? Yes ( ) Amount / day _________ No ( ) How long have you stopped? __________

I, _______________________________________________, Id ______________________, declare for all legal purposes that the information on my state of health is true and that I have omitted nothing in this questionnaire. I also agree to participate in the research project “Evaluation of the effects of photobiomodulation on orthodontic movement of molar verticalization with mini-implant: a randomized, double-blind pilot study”, knowing that I will only perform orthodontic movement of molar verticalization and, if necessary, I will be referred for other dental treatments, such as oral rehabilitation with prosthesis or implants.

Signature: _________________________________________ Date ____ / _______ / _______
