# Supplementary material for: Evaluation of the effects of photobiomodulation on orthodontic movement of molar verticalization with mini-implant: A randomized double-blind protocol study
Source: Medicine (Baltimore). 2020 Mar 27;99(13):e19430. doi: 10.1097/MD.0000000000019430 (PMC7220149; doi:10.1097/MD.0000000000019430)
Supplement: Supplemental Digital Content [file medi-99-e19430-s003.docx]

**Annex 3: OHIP-14 Questionnaire**

Answer the following questions by ticking the box that best fits your situation:

| **Questions** | **Never** | **Almost never** | **Sometimes** | **Oftentimes** | **Always** |
| --- | --- | --- | --- | --- | --- |
| 1. Do you have difficulty pronouncing a few words or speaking due to problems with your teeth, mouth or dental prosthesis? |  |  |  |  |  |
| 1. Do you feel your taste worsened due to problems with your teeth, mouth or dental prosthesis? |  |  |  |  |  |
| 1. Have you had pain in your mouth or teeth? |  |  |  |  |  |
| 1. Do you have difficulty eating any food due to problems with your teeth, mouth or dental prosthesis? |  |  |  |  |  |
| 1. Do you feel inhibited because of your teeth, mouth or dental prosthesis? |  |  |  |  |  |
| 1. Have you been feeling tense because of problems with your teeth, mouth or dental prosthesis? |  |  |  |  |  |
| 1. Your diet has been unsatisfactory due to problems with your teeth, mouth or dental prosthesis? |  |  |  |  |  |
| 1. Have you interrupted your meals due to problems with your teeth, mouth or dental prosthesis? |  |  |  |  |  |
| 1. Do you have difficulty relaxing due to problems with your teeth, mouth or dental prosthesis? |  |  |  |  |  |
| 1. Have you been embarrassed due to problems with your teeth, mouth or dental prosthesis? |  |  |  |  |  |
| 1. Have you been irritated with others due to problems with your teeth, mouth or dental prosthesis? |  |  |  |  |  |
| 1. Have you had difficulty performing your daily work due to problems with your teeth, mouth or dental prosthesis? |  |  |  |  |  |
| 1. Have you felt less satisfying life due to problems with your teeth, mouth or dental prosthesis? |  |  |  |  |  |
| 1. Have you been totally unable to meet your obligations due to problems with your teeth, mouth or dental prosthesis? |  |  |  |  |  |
